# Increasing the performance of Passion fruit (*Passiflora edulis*) seedlings by LED light regimes

**DOI:** 10.1038/s41598-021-00103-1

**Published:** 2021-10-25

**Authors:** Dangdi Liang, Ahmed Fathy Yousef, Xiaoxia Wei, Muhammad Moaaz Ali, Weijun Yu, Liuqing Yang, Ralf Oelmüller, Faxing Chen

**Affiliations:** 1 College of Horticulture, Fujian Agriculture and Forestry University, Fuzhou, 350002 China; 2 Fruit Research Institute, Fujian Academy of Agricultural Sciences, Fuzhou, 350013 China; 3 Department of Horticulture, College of Agriculture, University of Al-Azhar (Branch Assiut), Assiut, 71524 Egypt; 4 Matthias Schleiden Institute, Plant Physiology, Friedrich-Schiller-University Jena, Dornburgerstr. 159, 07743 Jena, Germany

**Keywords:** Plant sciences, Environmental sciences

## Abstract

Due to progress in the industrial development of light-emitting diodes (LEDs), much work has been dedicated to understanding the reaction of plants to these light sources in recent years. In this study, the effect of different LED-based light regimes on growth and performance of passion fruit (*Passiflora edulis*) seedlings was investigated. Combinations of different light irradiances (50, 100, and 200 µmol m^−2^ s^−1^), quality (red, green, and blue light-emitting LEDs), and photoperiods (10 h/14 h, 12 h/12 h and 14 h/10 h light/dark cycles) were used to investigate the photosynthetic pigment contents, antioxidants and growth traits of passion fruit seedlings in comparison to the same treatment white fluorescent light. Light irradiance of 100 µmol m^−2^ s^−1^ of a 30% red/70% blue LED light combination and 12 h/12 h light/dark cycles showed the best results for plant height, stem diameter, number of leaves, internode distance, and fresh/dry shoot/root weights. 14 h/10 h light/dark cycles with the same LED light combination promoted antioxidant enzyme activities and the accumulation of phenols and flavonoids. In contrast, lower light irradiance (50 µmol m^−2^ s^−1^) had negative effects on most of the parameters. We conclude that passion fruit seedlings' optimal performance and biomass production requires long and high light irradiances with a high blue light portion.

## Introduction

Fruits are widely recognized as a vital part of a healthy diet, and their regular intake may help to prevent a variety of ailments^1–3^. Optimal fruit production, permanent availability worldwide, and affordability are global issues to optimize human´s health. Passion fruit (*Passiflora edulis*) is an attractive, nutritious fruit crop highly appreciated for fresh consumption and industrial purposes because of its diverse uses for juice, jelly, and ice cream products^4^. *P. edulis* is a perennial woody fruit vine that belongs to the Passifloraceae family, native to tropical America (Brazil)^5^, and bears hermaphrodite, solitary flowers which are located in the leaf axils. The fruit has a stiff, smooth, waxy dark purple or yellow-hued peel with faint, fine white flecks. The fruit is mostly loaded with a fragrant mass of double-walled, membranous sacs holding orange-colored pulpy fluid and up to 250 tiny, hard, dark brown to black pitted seeds on the inside. The fruit has high nutritional and medicinal value. It is a rich source of vitamin A and C and contains fair amounts of iron, potassium, sodium, magnesium, sulfur, and chlorides, and has dietary fiber and protein (reviewed in^6^). Fruits are eaten fresh or processed into products like jams, squash, juice, cakes, pies, and ice-cream.

Photosynthetic photon flux density (PPFD) sources suitable for indoor cultivation in controlled environments have boosted crop productivity in densely populated areas or plant growth facilities, such as greenhouses^7^. The spectral properties of irradiances sources must meet the physiological requirements of plants for photosynthesis and photomorphogenic development^8^. However, for each crop species in its environment, the optimal quality and irradiance sources have to be found, and there is a huge spectrum of physiological processes which respond differently to the irradiances sources in different crop species. Light-emitting diodes (LEDs) have great potential for horticultural applications due to energy efficiency, longevity, and flexibility of their application^9^. LEDs become more and more suitable in research and commercial agriculture under controlled conditions due to their low radiation, heat, and wide spectral adaptation^10^.

Brown, et al.^11^ and Tennessen, et al.^12^ described the advantage of LED sources for optimal plant growth. The ideal spectral distribution range promotes plant growth with optimum life span and light energy efficiency^13^. Many studies investigated LED effects on growth, development, morphology, and photosynthesis in different plants^14–18^, used a combination of fluorescent and LED sources^19^, replaced fluorescent light tubes with LEDs^20^, or utilized various LED combinations.

It has been reported that different color ratios (different light spectrums) strongly influence on plants’ physiological and developmental outcomes^21–24^. Other researches revealed the effect of light irradiance on plant growth and development^23,25–29^ and photoperiod^23,30^. However, little is known about the combined effect of light irradiance, quality, and photoperiod on plants’ growth, development, and physiological response.

In the present study, we used a number of physiological and protective traits of passion fruit (*Passiflora edulis* var. Golden No. 6) seedlings to optimize their growth under various light regimes. We exposed the seedlings to light from LEDs emitting different wavelengths with three different light irradiances and for 3 light/dark cycles and propose an optimal regime for plant performance.

## Results

### Growth parameters

**Figure 1:**
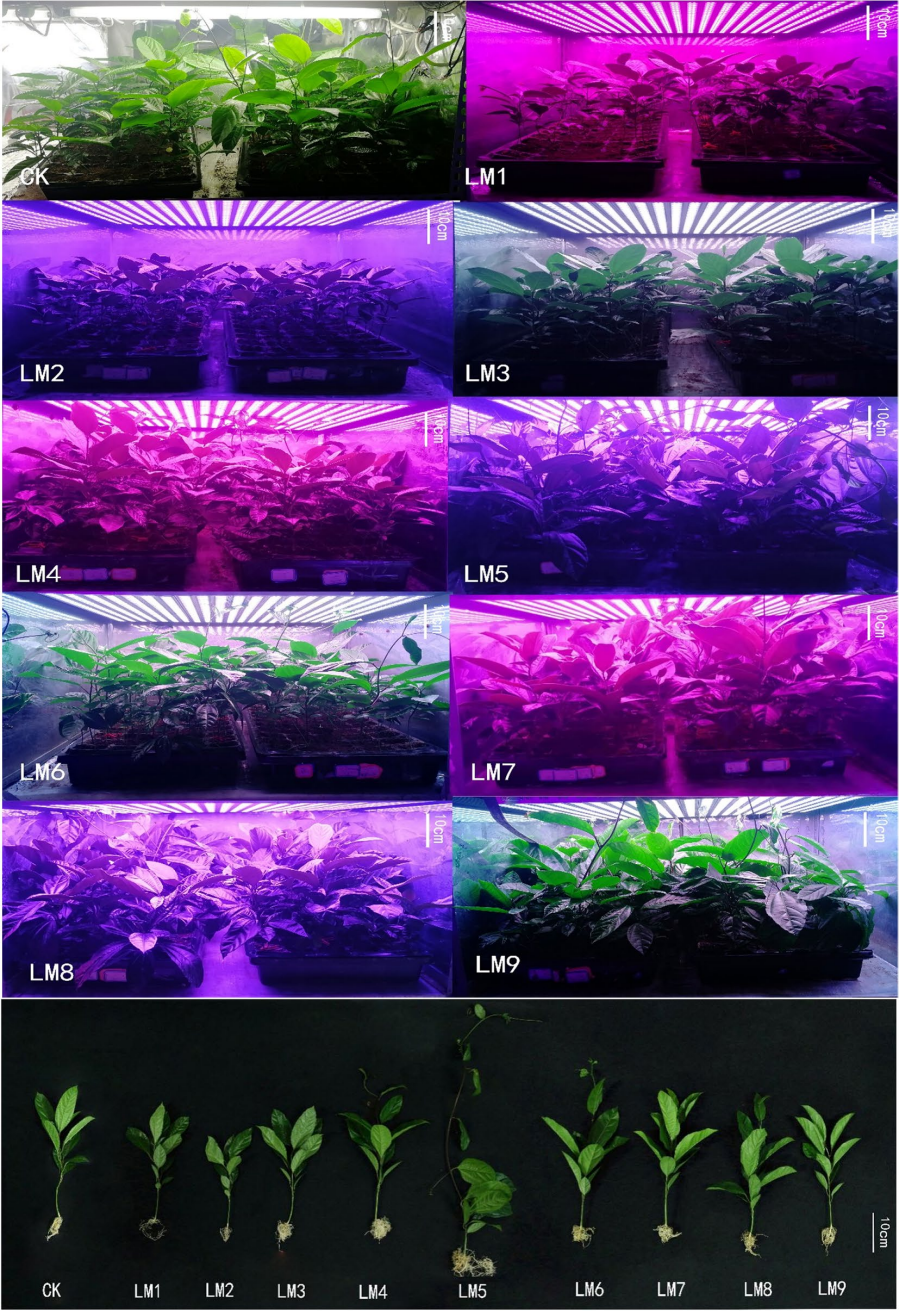
Passion fruit seedlings were grown 60 days under different LED light regimes. (Authors need to improve the quality of the superior figure. Images are compressed.

Table 1 and Fig. 1 show the effect of the different LED light regimes on the morphology and growth of the passion fruit seedlings. Table 1 demonstrates that the plant height, stem diameter, Number of leaves, internode distance, fresh shoot, and root weights, as well as dry shoot and root weights were the highest for the seedlings treated with the LM5 regime. The lowest values were observed for LM2, except for the root fresh weight. Although not statistically different to the other treatments, the leaf area of the seedlings was the highest with LM7 and the water content of the plants with LM3.

**Table 1:** Effect of different LED light regimes on plant morphology and growth characteristics of passion fruit seedlings.

Treatments	Plant height (cm)	Stem diameter (mm)	Leaf area (cm^2^)	Leaves no.	Internode distance (mm)	Fresh weight		Dry weight		Water content %
						Shoot (g.)	Root (g.)	Shoot (g.)	Root (g.)	
CK	28.8 ± 1.3 cd	2.91 ± 0.26bcd	48.89 ± 1.94ab	10.2 ± 0.58bcd	16.1 ± 1.1bc	6.55 ± 1.26bcd	1.12 ± 0.12bc	1.11 ± 0.30bcd	0.11 ± 0.03bc	84.48 ± 1.46bc
LM1	25.2 ± 0.6 cd	2.29 ± 0.03de	33.35 ± 3.31c	8.6 ± 0.40de	11.6 ± 0.3c	3.18 ± 0.13de	0.28 ± 0.02c	0.39 ± 0.02d	0.04 ± 0.00c	87.61 ± 0.10ab
LM2	22.5 ± 0.8d	2.11 ± 0.11e	32.91 ± 0.83c	8.4 ± 0.24e	11.5 ± 0.8c	2.28 ± 0.24e	0.45 ± 0.11bc	0.31 ± 0.00d	0.03 ± 0.01c	87.60 ± 0.42ab
LM3	26.7 ± 0.3 cd	2.62 ± 0.05cde	41.36 ± 3.08bc	8.8 ± 0.20cde	11.8 ± 0.4c	4.45 ± 0.59cde	0.52 ± 0.10bc	0.56 ± 0.09 cd	0.04 ± 0.01bc	87.85 ± 1.18a
LM4	42.2 ± 3.0b	3.44 ± 0.12b	46.64 ± 1.30ab	10.4 ± 0.24bc	23.5 ± 1.7bc	8.33 ± 0.21bc	1.31 ± 0.07bc	1.55 ± 0.16bc	0.16 ± 0.02 bc	82.12 ± 1.93c
LM5	69.0 ± 6.7a	4.11 ± 0.23a	46.24 ± 1.21ab	12.4 ± 0.60a	71.5 ± 9.7a	17.46 ± 1.89a	2.92 ± 0.46a	3.41 ± 0.55a	0.37 ± 0.07a	81.61 ± 0.90c
LM6	32.0 ± 2.2 cd	3.08 ± 0.10bc	41.10 ± 1.36bc	10.2 ± 0.37bcd	19.1 ± 1.8bc	8.03 ± 0.16bc	1.27 ± 0.01bc	1.08 ± 0.05bcd	0.14 ± 0.01bc	86.93 ± 0.25ab
LM7	27.9 ± 1.2 cd	3.42 ± 0.08b	54.54 ± 2.16a	9.4 ± 0.40bcde	18.7 ± 2.6bc	7.54 ± 0.78bc	1.41 ± 0.16b	1.84 ± 0.18b	0.18 ± 0.02b	77.33 ± 1.05d
LM8	36.4 ± 1.7bc	3.34 ± 0.22b	52.59 ± 2.78a	10.8 ± 0.20b	28.9 ± 2.9b	9.93 ± 0.38b	1.21 ± 0.17bc	1.77 ± 0.09b	0.15 ± 0.01bc	82.84 ± 0.41c
LM9	28.4 ± 0.5 cd	2.97 ± 0.24bcd	51.06 ± 1.93a	9.4 ± 0.24bcde	18.8 ± 0.5bc	6.30 ± 1.23bcd	1.11 ± 0.37bc	1.28 ± 0.31bcd	0.14 ± 0.05bc	81.17 ± 0.91c

Same letters indicate non-significant difference among treatments according to Duncan’s multiple range test (P ≤ 0.05). Vertical bars indicate average ± standard error (n = 4, 5 plants per replicate).

Table 2 shows the summary of the results based on the orthogonal array design. The optimal growth characteristics in response to the three factors (light irradiance, red-blue light ratio, and duration of LED light regimes) was A_2_B_2_C_3_, which indicated that the optimal light irradiance is 100 µmol m^−2^ s^−1^, from light with a R30:B70 ratio, and a photoperiod of 14 h light and 10 h dark. However, the best results for the leaf areas (A_2_B_1_C_2_) and water contents (A_1_B_3_C_3_) were obtained for different conditions.

**Table 2:** Results of the range and ANOVA of the L9 (3^3^) matrix for the influence of combined irradiances of LEDs light (A), light spectral ratios (B), and photoperiod (C) on growth characteristics of passion fruit seedlings.

Value		Plant height (cm)		Stem diameter (mm)		Leaf area (cm^2^)		Leaves no.		Inter-node distance (mm)		Fresh weight				Dry weight				Water content %	
												Shoot (g.)		Root (g.)		Shoot (g.)		Root (g.)			
		*R*	*P*	*R*	*P*	*R*	*P*	*R*	*P*	*R*	*P*	*R*	*P*	*R*	*P*	*R*	*P*	*R*	*P*	*R*	*P*
Factors	A	22.93	< .0001	1.20	< .0001	16.86	< .0001	2.40	< .0001	26.34	< .0001	7.97	< .0001	1.42	< .0001	1.59	< .0001	0.19	< .0001	7.24	< .0001
	B	13.60	0.0003	0.30	0.1094	0.93	0.8058	1.07	0.0027	20.76	< .0001	3.63	0.0016	0.56	0.0211	0.86	0.0053	0.08	0.0477	2.96	0.0061
	C	10.17	0.0021	0.54	0.0004	5.03	0.0302	0.80	0.0449	16.08	0.0006	4.18	0.0013	0.70	0.0042	0.89	0.0015	0.09	0.0085	3.53	0.0013
ELF		A > B > C		A > C > B		A > C > B		A > B > C		A > B > C		A > C > B		A > C > B		A > C > B		A > C > B		A > C > B	
BCm		A_2_B_2_C_3_		A_2_B_2_C_3_		A_2_B_1_C_2_		A_2_B_2_C_3_		A_2_B_2_C_3_		A_2_B_2_C_3_		A_2_B_2_C_3_		A_2_B_2_C_3_		A_2_B_2_C_3_		A_1_B_3_C_3_	

Where: Range value (R) – the range of difference between the maximum and minimum average; ELF – The most influential level factors on the parameter gradually; BCm – The best level combination for each parameter; (*P*-value) – ANOVA analysis of variance.

ANOVA analysis of the data is presented in Table 2 and shows that most of the differences are significant (*p* ˂ 0.05), except for the factor B on stem diameter and leaf area.

### Pigments contents

**Figure 2:**
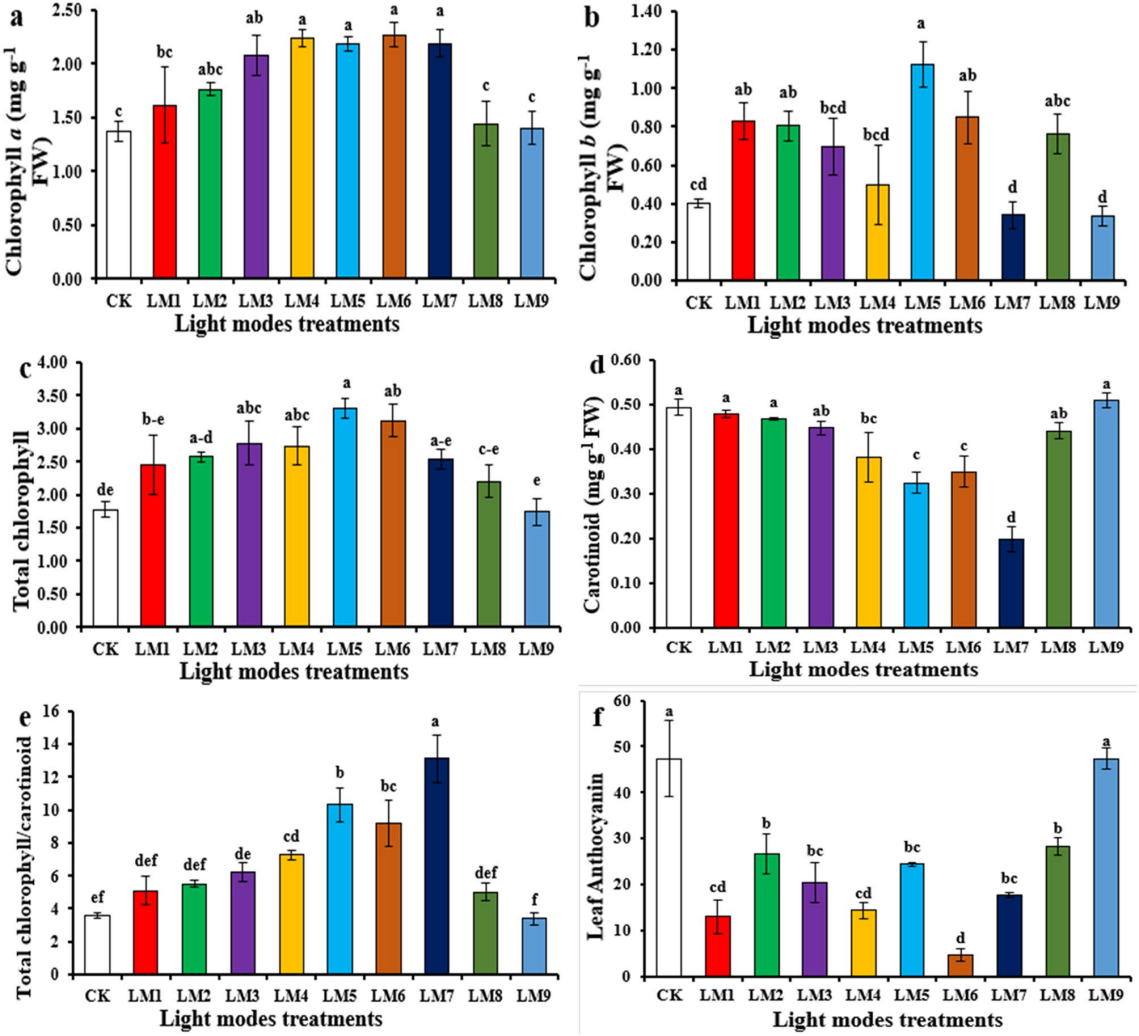
Effect of different LED light regimes on Chl *a* (**a**), Chl *b* (**b**), total Chl (**c**), carotenoid (**d**), ratio of total Chl to carotenoid (**e**), and anthocyanin contents (**f**) in leaves of passion seedlings. Each column represents the means of four replicates; different letters on similar columns indicate significant differences using Duncan's multiple range test at p ≤ 0.05.

**Figure 3:**
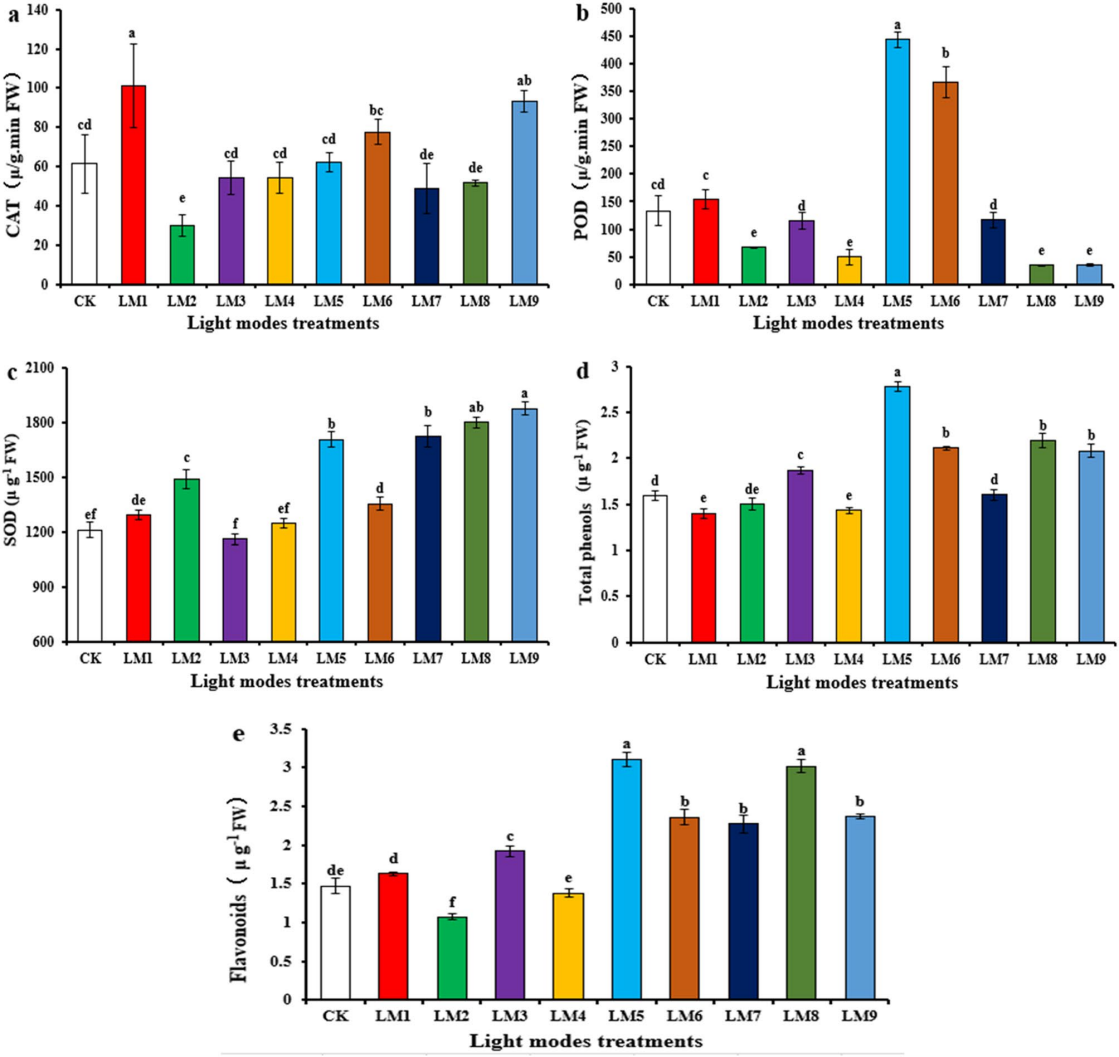
Effect of modes LED light on CAT (**a**), POD (**b**), SOD (**c**), Total phenols (**d**), and Flavonoids contents (**e**) in leaves of passion seedlings. Each column represents the means of four replicates; different letters on similar columns indicate significant differences using Duncan's multiple range test at p ≤ 0.05.

Next, we investigated whether the optimal light conditions obtained for plant growth and development are the same for accumulation of the pigments in the leaves (Fig. 2). As photosynthetic pigments, we assayed Chl *a*, Chl *b,* and the total carotenoid pool. Anthocyanin was chosen for a stress-related pigment which accumulates in the vacuole. Treatments with the light regimes LM3- LM7 gave almost identical results for the accumulation of Chl *a* (Fig. 2a), whereas the amounts of Chl *b* as well as of the total carotenoid pool were significantly lower under LM7, i.e., high light irradiance applied for a long period of time and with a high R portion (Fig. 2b,d). Since the leaf area under LM7 is the highest, the combination of these two results indicates that plants growing under LM7 can convert the photosynthetic energy quite efficiently into biomass production, which allows them to decrease their Chl *b* and car levels. Compared with CK treatment, the levels of total chlorophyll in the leaves of seedlings were higher with LM5 than with the other regimes of LED light, with LM2, LM3, LM4, LM6, and LM7 showing no statistical difference (Fig. 3c). The ratio of total chlorophyll to carotenoid was higher with LM7 than the other LED light regimes, while LM9 mode showed the lowest ratio (Fig. 3e).

Furthermore, anthocyanin is a stress pigment^31,32^, and its accumulation is more efficiently stimulated by cryptochrome than phytochromes^33^. Consequently, short illuminations with low light irradiances containing a high red-light portion (LM6) lead to the lowest anthocyanin accumulation (Fig. 2f). Moreover, since the amount of anthocyanin accumulating under LM7 is average, this light regime does not induce stress responses.

On the other hand, according to the R-values, the order of influence of the three factors on pigments contents of Passion fruit seedlings was observed in this study (Table 3). Table 3 shows that the order of impact of the three factors on chlorophyll a, chlorophyll b, total chlorophyll, carotenoid, total chlorophyll/carotenoid, and anthocyanin was (A > C > B), (A > B > C), (A > C > B), (C > A > B), (C > A > B), and (A > C > B), respectively.

**Table 3:** Results of the range and ANOVA of the L9 (3^3^) matrix for the influence of combined irradiances of LEDs light (A), light spectral ratios (B), and photoperiod (C) on Chl contents and antioxidants of passion fruit seedlings.

Value		Chl a		Chl b		Total Chl		Carotenoid		Total Chl/ Carotenoid		Anthocyanin		CAT		POD		SOD		Total phenols		Flavonoids	
		*R*	*P*	*R*	*P*	*R*	*P*	*R*	*P*	*R*	*P*	*R*	*P*	*R*	*P*	*R*	*P*	*R*	*P*	*R*	*P*	*R*	*P*
Factors	A	0.55	0.0070	0.343	0.0057	0.89	0.0017	0.11	0.0066	3.33	0.0224	16.67	0.0005	2.81	0.9553	224.54	< .0001	485.33	< .0001	0.52	< .0001	1.01	< .0001
	B	0.22	0.3958	0.341	0.0070	0.15	0.7390	0.08	0.0491	2.22	0.1408	11.44	0.0091	26.98	0.0476	74.76	0.1174	244.00	0.0010	0.68	< .0001	0.64	0.0001
	C	0.38	0.0501	0.27	0.0435	0.52	0.0664	0.13	0.0023	4.48	0.0017	14.11	0.0024	21.84	0.1114	174.25	0.0005	56.33	0.5768	0.41	0.0010	0.83	< .0001
ELF		A > C > B		A > B > C		A > C > B		C > A > B		C > A > B		A > C > B		B > C > A		A > C > B		A > B > C		B > A > C		A > C > B	
BCm		A_2_B_3_C_1_		A_2_B_2_C_3_		A_2_B_2_C_3_		A_3_B_3_C_2_		A_3_B_1_C_3_		A_3_B_3_C_2_		A_1_B_1_C_1_		A_2_B_2_C_3_		A_3_B_3_C_2_		A_2_B_2_C_3_		A_2_B_2_C_3_	

Where: Range value (R) – the range of difference between the maximal and minimal average; ELF – The most influential level factors on the parameter gradually; BCm – The best level combination for each parameter; (P-value) – ANOVA analysis of variance.

Based on the average of pigment contents derived from three factors at each level, the A_2_B_3_C_1_ and A_3_B_1_C_3_ were the best combinations gave the highest chlorophyll a and total chlorophyll/carotenoid, respectively, which indicated that the maximum of chlorophyll a and total chlorophyll/carotenoid presented at (irradiance 200 µmol m^−2^ s^−1^ + ratio (R70:G0:B30) + photoperiod 14 h/10 h) and (irradiance 100 µmol m^−2^ s^−1^ + ratio (R30:G0:B70) + photoperiod 14 h/10 h), respectively (Table 4). The best combination of different factors with the levels for the highest chlorophyll b and total chlorophyll was A_2_B_2_C_3_, which indicated that the maximum of these parameters presented at (irradiance 100 µmol m^−2^ s^−1^ + ratio (R30:G0:B70) + photoperiod 14 h/10 h). In contrast, the highest carotenoid and anthocyanin was A_3_B_3_C_2_, which indicated that the maximum of these parameters presented at (irradiance 200 µmol m^−2^ s^−1^ + ratio (R50:G20:B30) + photoperiod 12 h/12 h).

**Table 4:** Factors and levels of orthogonal experimental design.

Levels	Factors					
	A		B			C
1	50 ± 2		R70:G0:B30			10 h /14 h
2	100 ± 2		R30:G0:B70			12 h /12 h
3	200 ± 2		R50:G20:B30			14 h /10 h

Combinations of light modes using orthogonal test design						
Light modes	A	B	C	Layout of the L9 (3^3^) matrix		
				Levels A	Levels B	Levels C
LM1	1 (50 ± 2)	1 (R70:G0:B30)	1 (10 h /14 h)	1	1	1
LM2	1 (50 ± 2)	2 (R30:G0:B70)	2 (12 h /12 h)	1	2	2
LM3	1 (50 ± 2)	3 (R50:G20:B30)	3 (14 h /10 h)	1	3	3
LM4	2 (100 ± 2)	1 (R70:G0:B30)	2 (12 h /12 h)	2	1	2
LM5	2 (100 ± 2)	2 (R30:G0:B70)	3 (14 h /10 h)	2	2	3
LM6	2 (100 ± 2)	3 (R50:G20:B30)	1 (10 h /14 h)	2	3	1
LM7	3 (200 ± 2)	1 (R70:G0:B30)	3 (14 h /10 h)	3	1	3
LM8	3 (200 ± 2)	2 (R30:G0:B70)	1 (10 h /14 h)	3	2	1
LM9	3 (200 ± 2)	3 (R50:G20:B30)	2 (12 h /12 h)	3	3	2
CK	100 ± 2	–	12 h /12 h	–	–	–

Where: *Factor A* Photon flux density (μmol m^−2^ s^−1^), *Factor B* Light spectral ratios Red: Green: Blue; *Factor C* Photoperiod Light/Dark.

ANOVA (Table 3) showed that these three factors were significant effects on the plant morphology and the growth performance parameters of passion fruit seedlings (*p* ˂ 0.05), excepted factor B on chlorophyll a, total chlorophyll, and total chlorophyll/carotenoid had no significant effects.

### Antioxidant enzymes

The antioxidant enzyme activities for CAT, POD, and SOD, as well as the total phenol and flavonoid contents in leaves of passion fruit seedlings under different LEDs light treatments are shown in Fig. 3. The CAT activity was the highest in seedlings exposed to LM1, followed by LM9, while the activity was the lowest in seedlings exposed to LM2 (Fig. 3a). Interestingly, the results for the POD activity differed substantially: LM5 seedlings showed the highest and LM8 the lowest enzyme activity, and the LM8 treatment was comparable to those with LM2, LM4, and LM9 (Fig. 3b). The SOD activity was the highest in LM9 seedlings (Fig. 3c). The total phenol and flavonoid contents were the highest in LM5 seedlings (Fig. 3d,e). LM4 treatment resulted in the lowest total phenols content (Fig. 3d) and the LM2 treatment in the lowest flavonoids content (Fig. 3e).

Evaluation of the data showed that the three factors have quite different influences on the five antioxidant parameters with CAT (B > C > A), POD (A > C > B), SOD (A > B > C), total phenols (B > A > C), and flavonoids (A > C > B) (Table 4). The highest induction for POD, total phenols, and flavonoids was observed for A_2_B_2_C_3_, i.e. (100 µmol m^−2^ s^−1^ light of R30:G0:B70 ratio with a 14 h/10 h photoperiod), while the highest CAT was A_1_B_1_C_1_, which indicated that the maximum of CAT presented at (irradiance 50 µmol m^−2^ s^−1^ + ratio (R70:G0:B30) + photoperiod10h/14 h). The A_3_B_3_C_2_ was the best combination gave the highest SOD, which indicated that the maximum of SOD presented at (irradiance 200 µmol m^−2^ s^−1^ + ratio (R50:G20:B30) + photoperiod 12 h/12 h). However, when individual enzyme activities and pigment contents were considered, the optimal conditions were quite different from the average value, although ANOVA (Table 4) analyses showed that most of the differences were significant (*p* ˂ 0.05), excepted factor A and C on CAT, and C on SOD.

## Discussion

Light quality has a significant impact on the efficiency of photosynthesis and the morphological and physiological properties of the plant. In this study we tried to find the optimal light quality and quantity for biomass production and development of passion fruit seedlings. A literature survey demonstrates that the optimal light conditions for plants differ substantially^34,35^. The spectral distribution, illumination period and light irradiance have different effects on the efficiency of photosynthesis^36^, and the activation of the photoreceptors in different plant species^37^. The necessity of this study becomes important when our data on passion fruit are compared with those for agriculturally important plant species. In our experiments, passion fruit seedlings responded strongly to LM5 (100 µmol m^−2^ s^−1^; R30:G0:B70; 14 h/10 h) with regard to optimal plant height, stem diameter, leaves number, internode distance as well as shoot/root biomass (Table 1). Naznin, et al.^38^ and Yang, et al.^39^ investigated in pepper seedlings and Yang et al.^40^ tomato seedlings, and they found that in particular the red LED light was important for producing strong seedlings. Also, Yousef and coauthors showed that a combination of R and B LED light with a high R portion was effective in producing vigorous grafted tomato seedlings^28,29^. In most studies a combination of R and B light was more effective in promoting plant growth and development than the use of one LED source alone. For instance, cucumber seedlings grown under equal amounts of R and B light have higher yields compared to seedlings grown under R light only^41^. Yousef and co-authors extended the study to factory tomatoes and showed that illumination with 100 μmol m^−2^ s^−1^ 70% R and 30% B resulted in the optimal growth of the plants^42^. Moreover, the barrier tissue cells in the leaves were particularly well developed and the spongy tissue cells were arranged in an orderly manner^43^. It appears that blue light is important for passion fruit growth suggesting that activation of the cryptochrome system has a strong influence on its development.

The Chl content affects the photosynthetic ability and reduced carbon production^44,45^. Moreover, Chl biosynthesis is controlled by the quality of light, and many studies showed the important role of blue light^40,46–48^. Furthermore, ambient light conditions and stress can have profound effects on the Chl *a* and *b* levels. Our results showed that LM6 was the best light treatment for Chl *a* accumulation*,* followed by LM4, LM7, and LM5 treatments (Fig. 2a), whereas LM5 was optimal for Chl *b,* and LM9 for carotenoid and anthocyanin accumulation. This is in agreement with the findings of Yang et al.^40^ and Yousef et al.^42,49^ with tomato and pepper seedlings^38,39^. Furthermore, the authors observed that a mixture of red and blue LED lights induced higher levels of these pigments than white fluorescent light.

The benefits of light sources with enriched red and blue light were repeatedly shown for Chl accumulation, photosynthetic enzyme activity, stomatal aperture, and carbohydrate biosynthesis in plants^50–53^. Bondada et al*.*^50^ showed that red light increased the total Chl content and promoted photosynthesis rates but restricted the transport of carbohydrates from the leaves to the roots. In contrast, several studies showed that photomorphogenesis was more promoted by blue light, which increased Chl *a*/*b* ratios and facilitated stomatal opening^47,53,54^. Li et al.^55^ showed that besides increased photosynthesis per unit leaf area and a higher Chl *a/b* ratio, blue light also stimulated ribulose-1,5-bisphosphate carboxylase and phosphoenolpyruvate carboxylase activities, and promoted stomatal opening. Therefore, the red-blue light ratio has a strong influence on morphogenesis and ultimately biomass production. Red light facilitated cell division and expansion, resulting in increased leaf area and root elongation, while blue light inhibited these effects and reduced the leaf area and root elongation^56,57^. Besides light quality, also the irradiance and duration of illumination is important for yield; reduced photon irradiance in LM2 might be the main reason for the reduced leaf area and biomass production.

Besides photosynthetic parameters, the antioxidant status of the plant is important for fitness and agricultural yields. Antioxidants are involved in biotic and abiotic stress, including defense, oxidative damage, and free-radical scavenging^58^. Also, light stress plays a crucial role in the production, turnover, and destruction of antioxidants^59–61^. Red light increased the antioxidant activity in pea^62^ and *Dendrobium officinale* seedlings^63^, but also in *Eleutherococcus senticosus* somatic embryos^64^. However, the range in which different antioxidant enzymes react to stress differs substantially under different light conditions. Also Naznin, et al.^38^ showed that the antioxidant capacity of lettuce, spinach, cabbage, *Ocimum basilicum,* and *Capsicum annuum* was stimulated by red light, although various cultivars of the same plant can respond quite differently to different light situations^42,65^. Mengxi, et al.^66^ showed that blue light promoted antioxidant enzyme activities in *Oncidium*. Considering that plants have to decide to either invest in growth (photosynthesis) or defense, depending on environmental conditions, including light stress, optimal light sources for plants should balance these responses. Therefore, comparison of the effect of the different light regimes on photosynthesis-related and antioxidant-related responses is important for long-term benefits for the plant.

Plants can accumulate oxidative active chemicals under conditions with insufficient use of light energy during photosynthesis^67^. Antioxidant enzymes eliminate reactive oxygen species generated during (a)biotic stress including light stress, pathogen attack or senescence^68,69^. SOD removed H_2_O_2_ and O_2_^-^ generated during oxidative stress and lipid peroxidation; POD and CAT can remove H_2_O_2_ from peroxisomes and cytoplasm^70,71^. The highest antioxidant activities were found with LM1 (CAT; 50 µmol m^−2^ s^−1^; R70: G0: B30; 10 h/14 h), LM5 (SOD; 100 µmol m^−2^ s^−1^; R30:G0:B70; 14 h/10 h), and LM4 (POD, 100 µmol m^−2^ s^−1^; R70:G0:B30; 12 h/12 h) treatments. However, comparison with the other treatments suggest that neither the irradiance of LED light, the red-blue light ratio, nor the photoperiod caused high light stress to the passion fruit seedlings that resulted in an unusual upregulation of the antioxidant enzyme activities.

UV-B light stress affected polyphenol and flavonoid biosynthesis, since they absorb both visible and UV light to protect mesophyll cells from photooxidation^72,73^. This is important for plants in the field, but not under LED light.

In plants, there has been an increased biosynthesis of phenol and polyphenol compounds to help them cope with the multifarious abiotic and biotic stresses such as salinity, heavy metal, drought, temperature, UV lights, disease progression, etc.^74^. Cryptochromes and phytochromes, activated by blue, red and far-red LEDs have been intensively studied because they stimulate phenol and flavonoid accumulation in diverse plant species during the germination of sprouts^75,76^, in adult plants^77–79^, or during postharvest storage^80,81^. However, the effect of monochromatic LEDs for the induction of these secondary metabolites is dependent on numerous factors and has to be optimized for each plant species and growth condition. White, blue and red light stimulated the activities of the key enzymes of the shikimate and phenylpropanoid pathways, such as phenylalanine ammonia lyase^82^, chalcone isomerase and synthases, or the leucoanthocyanidin dioxygenase, flavanol synthase, dihydroflavonol 4-reductase and stilbene synthase involved in anthocyanin and flavonoid biosynthesis^83–85^. Not only biosynthesis genes, but also transcriptional activators and repressors play a role in the production of these phenolic compounds and respond to light^86^. Furthermore, greater production of phenylalanine rather than tryptophan was found to enhance the synthesis of phenolic compounds. This indicates that LEDs can regulate total phenol and total flavonoid concentration by promoting the expression of genes involved in the biosynthesis of the secondary compounds well as indirectly by boosting the precursor molecule^79^. In this study, the highest contents of total phenols and flavonoids was found with LM5 (100 µmol m^−2^ s^−1^; R30:G0:B70; 14 h/10 h light/dark cycles). Literature survey shows that only blue and red light sources were active, whereas green light was less effective^78,82,87^ or completely ineffective^81^. Loi, et al.^81^ did not find a significant increase in phenolic compounds in broccoli with green light, while Ouzounis, et al.^88^ found a stimulation under higher light irradiance, demonstrating that light irradiance is critical^79,89^. In general, higher light irradiances were more effective in activating light-responsive genes and the polyphenol content than lower light irradiances. Nonetheless, the findings are strongly dependent on the investigated plant species^77,90^, cultivars^91,92^, and timing of LED exposure^88^. Most notably, each polyphenolic compound reacts differently to different light qualities^75^. We also found that the CAT, SOD and POD activities responded differently to the different light regimes. Thus, a detailed analyses of the accumulation of antioxidant compounds is required for each plant species and cultivars, in particular when LEDs are used^93^.

## Material and methods

### Growth conditions and plant materials

**Figure 4:**
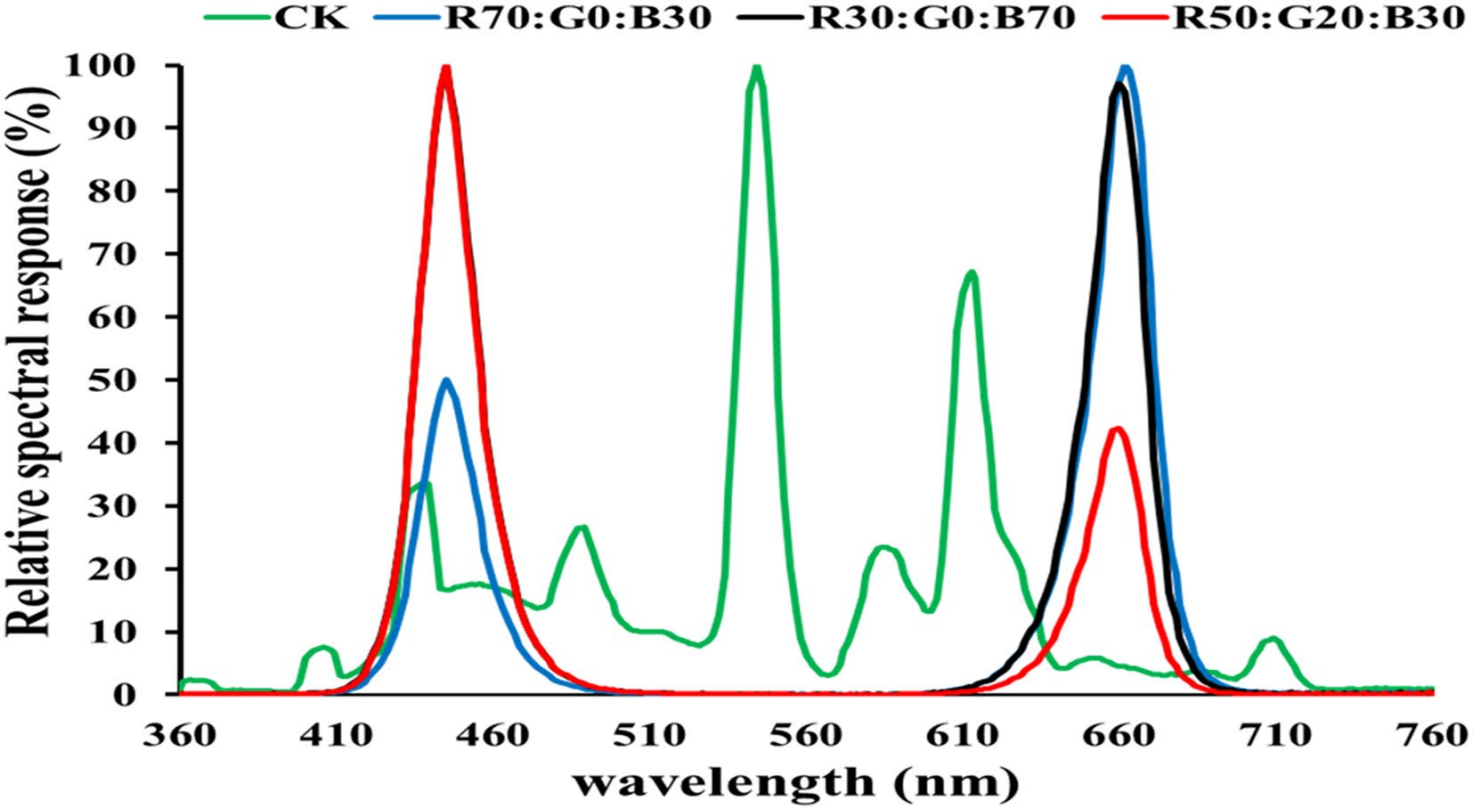
The treatments of LED light's spectrum distribution.

All experiments were conducted in LED light chambers at Fujian Agriculture and Forestry University, Fuzhou, Fujian, China. The manufacturer of the tested LED lamps is Kedao Technology Corporation (Huizhou, China) with the type of UH-BLDT0510. Our studies were complied with the relevant institutional, national, and international guidelines and legislation. The growth chambers at an optimum temperature, i.e., 27 ± 2 °C Day/23 ± 2 °C night, relative humidity was maintained at 65 ± 5% during the entire experimental procedures, and LED light's spectrum distribution as shown (Fig. 4).

The seeds were provided by the Fruit Research Institute, Fujian Academy of Agricultural Sciences. In August 2020, the fruits from the mature passion fruit variety ‘Golden No. 6’ was harvested, the seeds were cleaned with gauze bag, dried in a natural shade, and sealed in a Ziplock bag. To improve germination rate, the seeds were washed with distilled water, wrapped in wet gauze, placed in a Petri dish, and stored in an incubator at 35 °C. After 7 to 9 days, the percentage of germination is approximately 80%. The passion seeds were sown in 50-cell plug trays (28 cm width × 54 cm length × 8 cm height, Luoxi Plastic Products Co., Shandong, China) that was filled with commercial growing substrate (N_1_:P_1_:K_1_ ≥ 3%, organic matter ≥ 45%, pH 5.5–6.5). In total, 100 seedlings were grown in each box. Irrigation was provided for the seedlings daily or as required.

Seedlings were fertilized with water-soluble fertilizers (compound of fertilizer "N_20_: P_20_: K_20_ + TE", Ruierkang Co., Russia, and Stimufol Amino (compound fertilizers “N 25%, P 16%, K 12%, Fe 0.17%, amino acids 2%, Mo 0.001%, B 0.044%, Cu 0.085, Zn 0.03%, Mg 0.02%, Mn 0.085%, Co 0.01%, and EDTA”, Shoura Co., Egypt.)) two times per week through irrigation. Fertilization started one week after planting.

### Multiple-factor experiment design

The Orthogonal Experimental design method was used to analyze three factors with three levels each. As shown in Table 4, from the possible 27 treatments, 9 treatments (and a white fluorescent light control) were performed. Three light irradiances (50, 100, 200 μmol m^−2^ s^−1^; A1-A3) were given with red (R), green (G), and blue (B) LEDs filters (B1-B3) in 3 combinations (R:G:B in %: 70:0:30, 30:0:70, and 50:20:30 Fig. 4), and three photoperiods (C1-C3) (10/14 h, 12/12 h, and 14/10 light/dark cycles).

### Measurements and calculations

#### Measurement of growth and biomass parameters

Growth performance was estimated 60 days after sowing. We used a ruler to measure the plant height and the distance between nodes (cm), and a vernier caliper to measure the stem diameter (mm). The plant height was measured from the ground surface to the plant growth point; the stem diameter at the base of the stem; the node spacing between the 4th and 5th leaves. Leaf area (the third leaf below the top of the plant) was measured by LA-S Wanshen Plant Image Analyzer (Wanshen Testing Technology Co., Ltd., Hangzhou, China). The determination of the leaf dry material was obtained weighing the fresh leaves (washed and rinsed with deionized water), and the material was oven-dried (YH-9203A, Qingdao Yosion Labtech Co. Ltd., China) at 105 °C for 20 min, and drying continued at 70 °C, until a constant weight was reached.

#### Measurement of chlorophyll (Chl) content

Sixty days after sowing, the total Chl and carotenoid contents were determined from fresh medium-aged leaves with excluded the edges and veins of leaves (Fifth leaf from the top). Tissues of fresh leaves (0.2 g) were cut, ground well, suspended in 5 ml of 95% ethanol and filtered. The filtrate was made up to 25 ml by adding 95% ethanol. Absorbance of the filtered solution for Chl (Chl) *a*, Chl *b*, and carotenoid at 665 nm, 649 nm and 470 nm, respectively, was measured using a spectrophotometer (UV-5100B, Unico. Shanghai, China), while the Chl content was determined using the following equations^94^:

Chl *a* (mg g^−1^ FW) = (13.95 OD_665_ − 6.88 OD_649_) V/200 W

Chl *b* (mg g^−1^ FW) = (24.96 OD_649_ − 7.32 OD_663_) V/200 W

Total Chl (mg g^−1^ FW) = Chl *a* + Chl *b*

Car (mg g^−1^ FW) = (1000 OD_470_ − 2.05 Chl *a* − 114.80 Chl *b*) V/ (245 × 200 W)

Chl/Car ratio = (Chl *a* + Chl *b*) / Car

Where (Chl *a*) = chlorophyll *a*, (Chl *b*) = chlorophyll *b*, (Car.) = carotenoid, and (ToCh/Car.) = Total chlorophyll/ Carotenoid; (V) = volume (25 ml.) and (W) = sample weight (g).

#### Determination of anthocyanin

Sixty days after sowing, anthocyanin content was determined from fresh medium-aged leaves (Fifth leaf from the top) after removal of the veins. The plant material was cut into small pieces. To 0.1 g material, 5 mL 1% HCL-methanol solution was added, the solution was poured into a 5 mL centrifuge tube. The extraction took place in the dark until the tissue became white. The absorbance was measured at 530 and 600 nm and the anthocyanin content in leaves was calculated according to Cao, et al.^95^. The difference between the absorbance values at 530 nm and 600 nm represents the anthocyanin content, i.e. (OD_530_-OD_600_)/g.

#### Measurement of antioxidant enzyme activities

The superoxide dismutase (SOD) activity was determined by the nitrogen blue tetrazole (NBT) method of^96^. 0.1 g of frozen leaf (fresh medium-aged leaves Fifth leaf from the top) powder was weighed, 5 mL of phosphoric acid buffer (pH7.8) was added, and the slurry was centrifuged at 4000 rpm at 4° for 10 min. The supernatant was used for the enzyme assays. One unit of SOD caused inhibition of the photoreduction of NBT by 50%. The superoxide dismutase (SOD) activity was measured in the same extract by the increase in absorbance at 470 nm due to guaiacol oxidation activity and was quantified according to the method described by García-Triana, et al.^97^.

The peroxidase (POD) activity was determined by the guaiacol method^98^. 0.1 g of frozen leaf (Fifth leaf from the top) powder was weighed, resuspended in 10 mL phosphoric acid buffer (pH5.5) at 4 °C and centrifuged at 3000 rpm for 10 min. 0.1 mL enzyme extract was added to either 0.05 mol/L phosphoric acid buffer (2.9 mL), 2% H_2_O_2_ (1.0 mL), and 0.05 mol/L guaiacol (1.0 mL). Heat-treated extract (5 min) was used as control. The solution was incubated at 37 °C for 15 min, and then quickly transferred to an ice bath. 2.0 mL of 20% trichloroacetic acid was added to terminate the reaction. After centrifugation at 5000 rpm for 10 min, the absorbance was read at 470 nm according to Wang, et al.^99^.

The catalase (CAT) activity was determined by the ultraviolet absorption method^100^. 0.1 g of frozen fresh leaf (Fifth leaf from the top) powder was weighed and resuspended in 5 mL pre-cooled phosphoric acid buffer (pH 7.8, 1% polyvinylpyrrolidone). After accurate temperature adjustment to 5 °C for 10 min, the suspension was centrifuged at 4000 rpm for 15 min. The supernatant was used for the CAT assay by measuring the absorbance at 240 nm. All enzymes’ activities were expressed as unit’s min^−1^ g^−1^ sample.

#### Determination of total phenols and flavonoids

The total phenol and flavonoid contents were determined as previously described by Pourmorad, et al.^101^. To 0,1 g of frozen leaf (Fifth leaf from the top) powder 20 mL of 1% HCL-methanol solution was added to extract the pigments at 4 °C in the dark for 20 min. Total phenol and flavonoids content was determined by absorbance measurements at 280 and 325 nm, respectively.

#### Statistical analysis

The Orthogonal Experimental design method was used to determine the number of experiments to be conducted. All the data were subjected to one-way analysis of variance (ANOVA). Duncan's multiple range tests^102^ was used to test the significant difference between the means at 0.05 significance level using SPSS software (Version 16 SPSS Inc. Chicago, Illinois). The importance of the three factors for the measured parameters was assessed according to the effectiveness of each factor^103^ by the range value (*R*) using Excel 365 (v16.0). The most important impact factor has the greatest R-value. Adobe Illustrator software package version 23.0.3 was used to improve the quality of the images.

## Conclusion

Artificial light is very important for countries that do not have natural sunlight, especially LED light, because it consumes less electricity, produces less heat, and has a longer lifetime. In our research, we studied the effect of different combinations of light irradiances and qualities with different photoperiods on plant growth parameters, pigments contents, and some antioxidants. The best results were achieved with a mixture of red and blue light and a long photoperiod (either 12 h/12 h or 14 h/10 h light/dark cycles). The variation among the studied parameters underlines the importance of this study to understand which light regime is optimal for growth and development of passion fruit seedlings to balance growth and stress responses.

## References

[1] Winston C, Beck L (1999). Phytochemicals: Health protective effects. Can. J. Diet. Pract. Res..

[2] Ali MM, Yousef AF, Li B, Chen F (2021). Effect of environmental factors on growth and development of fruits. Trop. Plant Biol..

[3] Ali MM (2021). Influence of bagging on the development and quality of fruits. Plants.

[4] Zhang X (2021). Changes in the content of organic acids and expression analysis of citric acid accumulation-related genes during fruit development of yellow (Passiflora edulis f flavicarpa) and Purple (Passiflora edulis f edulis) Passion Fruits. Int. J. Mol. Sci..

[5] Knight, R. & Winters, H. in *Florida State Horticultural Society.* 412–418.

[6] Thokchom R, Mandal G (2017). Production preference and importance of passion fruit (Passiflora edulis): A review. J. Agric. Eng. Food Technol..

[7] Yeh N, Chung J-P (2009). High-brightness LEDs—Energy efficient lighting sources and their potential in indoor plant cultivation. Renew. Sustain. Energy Rev..

[8] Bula RJ (1991). Light-emitting diodes as a radiation source for plants. HortScience.

[9] Bourget CM (2008). An introduction to light-emitting diodes. HortScience.

[10] Morrow RC (2008). LED lighting in horticulture. HortScience.

[11] Brown CS, Schuerger AC, Sager JC (1995). Growth and photomorphogenesis of pepper plants under red light-emitting diodes with supplemental blue or far-red lighting. J. Am. Soc. Horticult. Sci..

[12] Tennessen DJ, Singsaas EL, Sharkey TD (1994). Light-emitting diodes as a light source for photosynthesis research. Photosynth. Res..

[13] Schubert EF, Kim JK (2005). Solid-state light sources getting smart. J Science.

[14] Folta KM, Maruhnich SA (2007). Green light: a signal to slow down or stop. J. Exp. Bot..

[15] Goins GD, Yorio NC, Sanwo M, Brown C (1997). Photomorphogenesis, photosynthesis, and seed yield of wheat plants grown under red light-emitting diodes (LEDs) with and without supplemental blue lighting. J. Exp. Bot..

[16] Kim H-H, Goins GD, Wheeler RM, Sager JC (2004). Stomatal conductance of lettuce grown under or exposed to different light qualities. Ann. Bot..

[17] Xiaoying L, Shirong G, Taotao C, Zhigang X, Tezuka T (2012). Regulation of the growth and photosynthesis of cherry tomato seedlings by different light irradiations of light emitting diodes (LED). Afr. J. Biotech..

[18] Elmardy NA (2021). Photosynthetic performance of rocket (*Eruca sativa* Mill.) grown under different regimes of light intensity, quality, and photoperiod. PLoS ONE.

[19] Robertson, J. Combination fluorescent and LED lighting system. (2007).

[20] Robertson, J. J. & Currie, R. M. LED replacement for fluorescent lighting. (2005).

[21] Loconsole D, Cocetta G, Santoro P, Ferrante A (2019). Optimization of LED lighting and quality evaluation of romaine lettuce grown in an innovative indoor cultivation system. Sustainability.

[22] Borowski E, Michałek S, Rubinowska K, Hawrylak-Nowak B, Grudzinski W (2015). The effects of light quality on photosynthetic parameters and yield of lettuce plants. Acta Sci. Polonorum.

[23] Yan Z, He D, Niu G, Zhai H (2019). Evaluation of growth and quality of hydroponic lettuce at harvest as affected by the light intensity, photoperiod and light quality at seedling stage. Sci. Hortic..

[24] Lin K-H (2013). The effects of red, blue, and white light-emitting diodes on the growth, development, and edible quality of hydroponically grown lettuce (Lactuca sativa L var capitata). Sc. Horticult..

[25] Fu Y (2017). Interaction effects of light intensity and nitrogen concentration on growth, photosynthetic characteristics and quality of lettuce (Lactuca sativa L. Var. youmaicai). Sci. Horticult..

[26] Nguyen T, Tran T, Nguyen Q (2019). Effects of light intensity on the growth, photosynthesis and leaf microstructure of hydroponic cultivated spinach (Spinacia oleracea L.) under a combination of red and blue LEDs in house. Int. J. Agric. Technol..

[27] Muneer S, Kim EJ, Park JS, Lee JH (2014). Influence of green, red and blue light emitting diodes on multiprotein complex proteins and photosynthetic activity under different light intensities in lettuce leaves (Lactuca sativa L). Int. J. Mol. Sci..

[28] Yousef AF (2021). Effects of light spectrum on morpho-physiological traits of grafted tomato seedlings. PLoS ONE.

[29] Yousef AF (2021). Light quality and quantity affect graft union formation of tomato plants. Sci. Rep..

[30] Kang JH, KrishnaKumar S, Atulba SLS, Jeong BR, Hwang SJ (2013). Light intensity and photoperiod influence the growth and development of hydroponically grown leaf lettuce in a closed-type plant factory system. Horticult. Environ. Biotechnol..

[31] Cirillo V (2021). Anthocyanins are key regulators of drought stress tolerance in tobacco. Biology.

[32] Zhu H (2017). Effects of low light on photosynthetic properties, antioxidant enzyme activity, and anthocyanin accumulation in purple pak-choi (Brassica campestris ssp. Chinensis Makino). PLoS ONE.

[33] Mancinelli AL, Rossi F, Moroni A (1991). Cryptochrome, phytochrome, and anthocyanin production. Plant Physiol..

[34] Olle M, Viršile A (2013). The effects of light-emitting diode lighting on greenhouse plant growth and quality. Agric. Food Sci..

[35] Zheng L, He H, Song W (2019). Application of light-emitting diodes and the effect of light quality on horticultural crops: A review. HortScience.

[36] Berg JM, Tymoczko JL, Stryer L (2002). Biochemistry.

[37] Brodersen CR, Vogelmann TC (2010). Do changes in light direction affect absorption profiles in leaves?. Funct. Plant Biol..

[38] Naznin MT, Lefsrud M, Gravel V, Azad MOK (2019). Blue light added with Red LEDs enhance growth characteristics, pigments content, and antioxidant capacity in lettuce, spinach, kale, basil, and sweet pepper in a controlled environment. Plants.

[39] Yang Z (2017). Plant growth and development of pepper seedlings under different photoperiods and photon flux ratios of red and blue LEDs. Trans. Chin. Soc. Agric. Eng..

[40] Yang X (2018). Response of photosynthetic capacity of tomato leaves to different LED light wavelength. Environ. Exp. Bot..

[41] Jeong HW (2020). Using light quality for growth control of cucumber seedlings in closed-type plant production system. Plants.

[42] Yousef AF (2021). Photosynthetic apparatus performance of tomato seedlings grown under various combinations of LED illumination. PLoS ONE.

[43] XiaoYing L, ShiRong G, ZhiGang X, XueLei J, Tezuka T (2011). Regulation of chloroplast ultrastructure, cross-section anatomy of leaves, and morphology of stomata of cherry tomato by different light irradiations of light-emitting diodes. HortScience.

[44] Curran PJ, Windham WR, Gholz HL (1995). Exploring the relationship between reflectance red edge and chlorophyll concentration in slash pine leaves. Tree Physiol..

[45] Gitelson AA, Gritz Y, Merzlyak MN (2003). Relationships between leaf chlorophyll content and spectral reflectance and algorithms for non-destructive chlorophyll assessment in higher plant leaves. J. Plant Physiol..

[46] Hoffmann AM, Noga G, Hunsche M (2015). High blue light improves acclimation and photosynthetic recovery of pepper plants exposed to UV stress. Environ. Exp. Bot..

[47] Sæbø A, Krekling T, Appelgren M (1995). Light quality affects photosynthesis and leaf anatomy of birch plantlets in vitro. Plant Cell, Tissue Organ Cult..

[48] Zheng L, Van Labeke M-C (2017). Long-term effects of red-and blue-light emitting diodes on leaf anatomy and photosynthetic efficiency of three ornamental pot plants. Front. Plant Sci..

[49] Yousef AF (2021). The influence of LEDs light quality on the growth pigments biochemical and chlorophyll fluorescence characteristics of tomato seedlings (*Solanum lycopersicum* L.). Fresenius Environ. Bull..

[50] Bondada BR, Syvertsen JP (2003). Leaf chlorophyll, net gas exchange and chloroplast ultrastructure in citrus leaves of different nitrogen status. Tree Physiol..

[51] McCree KJ (1971). The action spectrum, absorptance and quantum yield of photosynthesis in crop plants. Agric. Meteorol..

[52] Sager J, Smith W, Edwards J, Cyr K (1988). Photosynthetic efficiency and phytochrome photoequilibria determination using spectral data. Trans. ASAE.

[53] Senger H (1982). The effect of blue light on plants and microorganisms. Photochem. Photobiol..

[54] Baroli I, Price GD, Badger MR, von Caemmerer S (2008). The contribution of photosynthesis to the red light response of stomatal conductance. Plant Physiol..

[55] Li Q (2010). Effects of Light Quality on Growth and Phytochemical Accumulation of Lettuce and Salvia miltiorrhiza Bunge.

[56] Bugbee, B. in *VIII International Symposium on Light in Horticulture 1134* 1–12 (2016).

[57] Yun K, Shaohui W, Hongxiang S (2006). Effects of supplemental lighting with different light quality on the shoot growth of grape growing in greenhouse. J. Beijing Agric. Coll..

[58] Gupta DK, Palma JM, Corpas FJ (2018). Antioxidants and antioxidant enzymes in higher plants.

[59] Hasanuzzaman M (2019). Regulation of ascorbate-glutathione pathway in mitigating oxidative damage in plants under abiotic stress. Antioxidants.

[60] Biswas K (2020). Sustainable Agriculture in the Era of Climate Change.

[61] Kumar N, Singh H, Sharma SK (2020). Sustainable Agriculture in the Era of Climate Change.

[62] Wu M-C (2007). A novel approach of LED light radiation improves the antioxidant activity of pea seedlings. Food Chem..

[63] Wang Y (2017). Effects of different light qualities on seedling growth and chlorophyll fluorescence parameters of Dendrobium officinale. Biologia.

[64] Shohael A (2006). Effect of light on oxidative stress, secondary metabolites and induction of antioxidant enzymes in Eleutherococcus senticosus somatic embryos in bioreactor. Process Biochem..

[65] Cervantes L (2019). Light exposure affects fruit quality in different strawberry cultivars under field conditions. Sci. Hortic..

[66] Mengxi L, Zhigang X, Yang Y, Yijie F (2011). Effects of different spectral lights on Oncidium PLBs induction, proliferation, and plant regeneration. Plant Cell, Tissue Organ Cult..

[67] Foyer CH, Lelandais M, Kunert KJ (1994). Photooxidative stress in plants. Physiol. Plant..

[68] Molassiotis A, Dimassi K, Diamantidis G, Therios I (2004). Changes in peroxidases and catalase activity during in vitro rooting. Biol. Plant..

[69] Pastori GM, del Río LA (1997). Natural senescence of pea leaves (an activated oxygen-mediated function for peroxisomes). Plant Physiol..

[70] Dewir Y, Chakrabarty D, Ali M, Hahn E, Paek K (2006). Lipid peroxidation and antioxidant enzyme activities of Euphorbia millii hyperhydric shoots. Environ. Exp. Bot..

[71] Wu H (2016). Effect of different light qualities on growth, pigment content, chlorophyll fluorescence, and antioxidant enzyme activity in the red alga Pyropia haitanensis (Bangiales, Rhodophyta). Biomed. Res. Int..

[72] Xu J (2019). Response of bioactive phytochemicals in vegetables and fruits to environmental factors. Eur. J. Nutr. Food Saf..

[73] Sharma A (2019). Response of phenylpropanoid pathway and the role of polyphenols in plants under abiotic stress. Molecules.

[74] Tuladhar, P., Sasidharan, S. & Saudagar, P. in *Biocontrol Agents and Secondary Metabolites* 419–441 (2021).

[75] Acharya J, Rechner O, Neugart S, Schreiner M, Poehling H-M (2016). Effects of light-emitting diode treatments on Brevicoryne brassicae performance mediated by secondary metabolites in Brussels sprouts. J. Plant Dis. Protect..

[76] Nam TG, Kim D-O, Eom SH (2018). Effects of light sources on major flavonoids and antioxidant activity in common buckwheat sprouts. Food Sci. Biotechnol..

[77] Taulavuori K, Pyysalo A, Taulavuori E, Julkunen-Tiitto R (2018). Responses of phenolic acid and flavonoid synthesis to blue and blue-violet light depends on plant species. Environ. Exp. Bot..

[78] Kim E-Y, Park S-A, Park B-J, Lee Y, Oh M-M (2014). Growth and antioxidant phenolic compounds in cherry tomato seedlings grown under monochromatic light-emitting diodes. Horticult. Environ. Biotechnol..

[79] Wang P (2020). Exploration of the effects of different blue LED light intensities on flavonoid and lipid metabolism in tea plants via transcriptomics and metabolomics. Int. J. Mol. Sci..

[80] Amoozgar A, Mohammadi A, Sabzalian M (2017). Impact of light-emitting diode irradiation on photosynthesis, phytochemical composition and mineral element content of lettuce cv, Grizzly. Photosynthetica.

[81] Loi M (2019). Effect of different light-emitting diode (LED) irradiation on the shelf life and phytonutrient content of broccoli (Brassica oleracea L. var. italica). Food Chem..

[82] Wilawan N, Ngamwonglumlert L, Devahastin S, Chiewchan N (2019). Changes in enzyme activities and amino acids and their relations with phenolic compounds contents in okra treated by LED lights of different colors. Food Bioprocess Technol..

[83] Ahn S, Kim S, Yun H (2015). Inhibition of Botrytis cinerea and accumulation of stilbene compounds by light-emitting diodes of grapevine leaves and differential expression of defense-related genes. Eur. J. Plant Pathol..

[84] Gam DT (2020). LED lights promote growth and flavonoid accumulation of anoectochilus roxburghii and are linked to the enhanced expression of several related genes. Plants.

[85] Gupta SD, Pradhan S (2017). Regulation of gene expression by LED lighting. Light Emit. Diodes Agric..

[86] Liu J, Osbourn A, Ma P (2015). MYB transcription factors as regulators of phenylpropanoid metabolism in plants. Mol. Plant.

[87] Huang JY, Xu F, Zhou W (2018). Effect of LED irradiation on the ripening and nutritional quality of postharvest banana fruit. J. Sci. Food Agric..

[88] Ouzounis T, Razi Parjikolaei B, Fretté X, Rosenqvist E, Ottosen C-O (2015). Predawn and high intensity application of supplemental blue light decreases the quantum yield of PSII and enhances the amount of phenolic acids, flavonoids, and pigments in Lactuca sativa. Front. Plant.

[89] Samuolienė G (2017). Blue light dosage affects carotenoids and tocopherols in microgreens. Food Chem..

[90] Taulavuori K, Hyöky V, Oksanen J, Taulavuori E, Julkunen-Tiitto R (2016). Species-specific differences in synthesis of flavonoids and phenolic acids under increasing periods of enhanced blue light. Environ. Exp. Bot..

[91] Maroga GM, Soundy P, Sivakumar D (2019). Different postharvest responses of fresh-cut sweet peppers related to quality and antioxidant and phenylalanine ammonia lyase activities during exposure to light-emitting diode treatments. Foods.

[92] Son K-H, Oh M-M (2013). Leaf shape, growth, and antioxidant phenolic compounds of two lettuce cultivars grown under various combinations of blue and red light-emitting diodes. HortScience.

[93] Loi M, Villani A, Paciolla F, Mulè G, Paciolla C (2021). Challenges and opportunities of light-emitting diode (LED) as key to modulate antioxidant compounds in plants. A review. Antioxidants.

[94] Knight, S. & Mitchell, C. Enhancement of lettuce yield by manipulation of light and nitrogen nutrition. *HortScience: a publication of the American Society for Horticultural Science* **108**, 750–754 (1983). 11542284

[95] Cao J-K, Jiang W-B, Zhao Y-M (2007). Experimental Guide for Physiology and Biochemistry of Post-Harvest Fruits and Vegetables.

[96] Rao KM, Sresty T (2000). Antioxidative parameters in the seedlings of pigeonpea (Cajanus cajan (L.) Millspaugh) in response to Zn and Ni stresses. Plant Sci..

[97] García-Triana A, Zenteno-Savín T, Peregrino-Uriarte AB, Yepiz-Plascencia G (2010). Hypoxia, reoxygenation and cytosolic manganese superoxide dismutase (cMnSOD) silencing in Litopenaeus vannamei: effects on cMnSOD transcripts, superoxide dismutase activity and superoxide anion production capacity. Dev. Comp. Immunol..

[98] Nakano Y, Asada K (1981). Hydrogen peroxide is scavenged by ascorbate-specific peroxidase in spinach chloroplasts. Plant Cell Physiol..

[99] Wang L (2014). Regulation of POD activity by pelargonidin during vegetative growth in radish (*Raphanus sativus* L.). Sci. Horticult..

[100] Aebi H (1984). Catalase in vitro. Methods Enzymol.

[101] Pourmorad F, Hosseinimehr S, Shahabimajd N (2006). Antioxidant activity, phenol and flavonoid contents of some selected Iranian medicinal plants. Afr. J. Biotech..

[102] Duncan DB (1955). Multiple range and multiple F tests. J. Biometr..

[103] Roy R (1990). A Primer on Taguchi Method.

